# Deep Learning to Enhance Diagnosis and Management of Intrahepatic Cholangiocarcinoma

**DOI:** 10.3390/cancers17101604

**Published:** 2025-05-09

**Authors:** Charalampos Theocharopoulos, Achilleas Theocharopoulos, Stavros P. Papadakos, Nikolaos Machairas, Timothy M. Pawlik

**Affiliations:** 1 Second Department of Propaedeutic Surgery, Laiko General Hospital, School of Medicine, National Kapodistrian University of Athens, 11527 Athens, Greece; 2 Department of Electrical and Computer Engineering, National Technical University of Athens, 10682 Athens, Greece; 3 Department of Gastroenterology, Laiko General Hospital, National and Kapodistrian University of Athens, 11527 Athens, Greece; 4 Department of Surgery, The Ohio State University Wexner Medical Center and James Comprehensive Cancer Center, Columbus, OH 43210, USA

**Keywords:** deep learning, artificial intelligence, intrahepatic cholangiocarcinoma, hepatocellular carcinoma, convolutional neural networks, computer-aided diagnosis

## Abstract

Intrahepatic cholangiocarcinoma (iCCA) is associated with a poor prognosis and necessitates a multimodal, multidisciplinary approach from diagnosis to treatment to achieve optimal outcomes. A noninvasive preoperative diagnosis using abdominal imaging techniques can represent a clinical challenge. Given the differential response of iCCA to localized and systemic therapies compared with hepatocellular carcinoma and secondary hepatic malignancies, an accurate diagnosis is crucial. Deep learning (DL) models for image analysis have emerged as a promising adjunct for the abdominal radiologist, potentially enhancing the accurate detection and diagnosis of iCCA. Over the last five years, several reports have proposed robust DL models, which demonstrate a diagnostic accuracy that is either comparable to or surpasses that of radiologists with varying levels of experience. Recent studies have expanded DL applications into other aspects of iCCA management, including histopathologic diagnosis, the prediction of histopathological features, the preoperative prediction of survival, and the pretreatment prediction of responses to systemic therapy. We herein critically evaluate the expanding body of research on DL applications in the diagnosis and management of iCCA, providing insights into the current progress and future research directions. We comprehensively synthesize the performance and limitations of DL models in iCCA research, identifying key challenges that serve as a translational reference for clinicians.

## Simple Summary

Advancements in artificial intelligence are transforming medical diagnostics and treatment planning. Deep learning, a subset of AI, has demonstrated significant potential to analyze complex imaging and structured clinical data. Intrahepatic cholangiocarcinoma represents a challenging nosological entity from diagnosis to treatment. DL models have the potential to improve image-based iCCA diagnosis and optimize surgical and oncological decision-making. This manuscript aims to summarize and critically evaluate the present body of literature on DL approaches related to the diagnosis and management of iCCA.

## 1. Introduction

**Figure 1:**
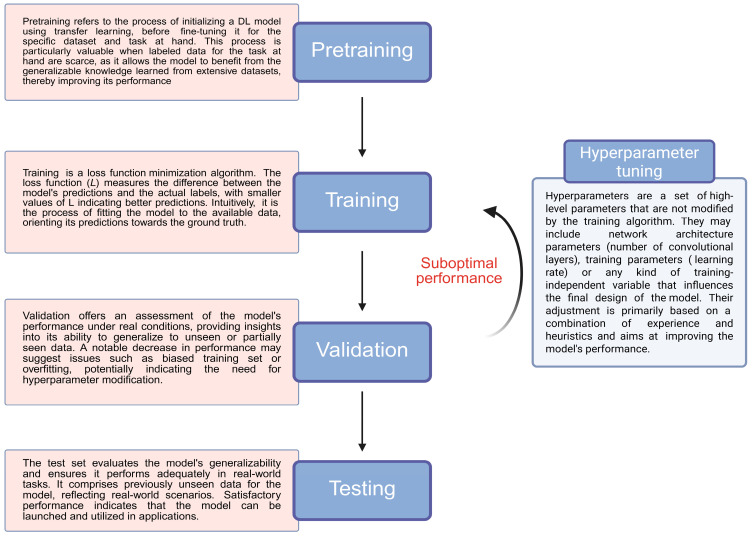
A summary of the main phases of developing a DL model.

**Figure 2:**
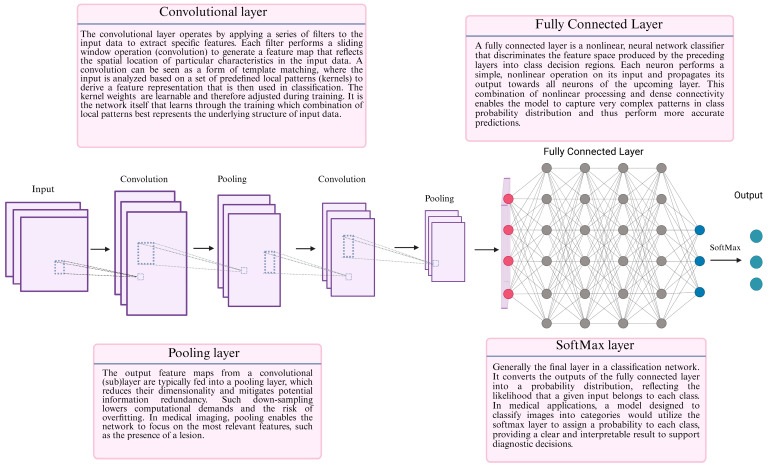
A schematic representation of a basic CCN architecture. A vanilla CNN architecture consists of alternating convolutional and pooling layers. These layers are followed by fully connected layers which integrate the extracted features for classification, concluding with a SoftMax layer that generates a probability distribution across the different classes.

The use of artificial intelligence (AI) models in healthcare has expanded dramatically over the past decade, evolving from research settings to the regulatory approval of numerous AI-enabled medical devices for routine clinical practice [1]. According to bibliometric data, healthcare-related AI publications more than doubled in number in 2023 versus 2021, reflecting the growing interest in the implementation of AI in medicine [2]. Medical imaging and upper and lower gastrointestinal endoscopy constitute the fields in which DL has particularly been utilized [3,4]. These trends in AI-related research largely depict the transition from first-generation rule-based systems to machine learning (ML) and, more recently, deep learning (DL) models [5]. Based on the data supplied, these models constantly evolve through autonomous learning, make informed decisions and predictions, as well as output results. DL models have been proven to be accurate in image analysis, classification, and segmentation tasks, outperforming ML due to their ability to automatically extract and learn hierarchical features from images [6]. DL models can be utilized in medical image interpretation through either computer-aided detection (CADe) or computer-aided diagnosis (CADx). CADe systems are developed to identify objects or lesions, while CADx systems are designed to classify the detected objects or lesions. The process of developing a robust DL model typically involves four main phases in a sequential order: pretraining, training, validation, and testing (Figure 1). The convolutional neural network (CNN) architecture provides the framework for DL models (Figure 2). The functional principles of CNN-building blocks have been previously reviewed and are beyond the scope of the current manuscript [7,8]. Table 1 provides an explanation of the main evaluation metrics used in the presented studies to facilitate comprehension and critical evaluation.

Intrahepatic cholangiocarcinoma (iCCA) is an aggressive malignancy with a grave prognosis that necessitates multimodal and multidisciplinary management. While playing a pivotal role in diagnosis, abdominal imaging modalities cannot always distinguish iCCA from other hepatic malignancies, particularly hepatocellular carcinoma, (HCC) with a high reproducibility and fidelity [9]. Given the differential response to systemic therapies and the different surgical management required for iCCA versus HCC, an accurate pretreatment diagnosis is important to optimize patient care. In turn, DL may have the potential to improve iCCA management by enhancing imaging-based diagnostic accuracy, facilitating the proper assignment of patients to the appropriate treatment algorithms. Several recent studies have proposed highly effective DL models with performances equal or superior to radiologists to diagnose iCCA (Table 2) [10,11,12]. Other efforts have evaluated DL models related to other aspects of iCCA management, including histopathologic diagnosis, the prediction of histopathological features, the preoperative prediction of survival, and the prediction of responses to systemic therapy (Table 3 and Table 4) [13,14,15,16]. DL models have also been investigated relative to surgical decision-making. For example, the preoperative assessment of iCCA typically involves determining resectability, evaluating vascular invasion, and estimating the tumor burden [17]. Emerging DL models have demonstrated the ability to predict tumor invasion patterns, lymph node metastases, and microvascular invasion (MVI) based on imaging and histopathologic features, allowing for even more precise surgical planning [13,15,18]. The ability to identify high-risk features preoperatively may influence decisions such as the need for extended hepatectomy, vascular resection and reconstruction, or the necessity for adjuvant therapies [19].

Given the important and emerging role of artificial intelligence in the clinical setting, we herein review the role of DL in the diagnosis and management of iCCA with a particular focus on the potential of DL to enhance imaging accuracy, surgical decision-making, and patient outcomes. By evaluating the performance and limitations of published models and discussing them within the clinical workflow of iCCA management, we offer an update on DL related to iCCA diagnosis and treatment, as well as highlight the direction of future efforts. This review aims to serve as a translational reference for researchers and clinicians interested in leveraging DL to improve patient care in iCCA.

To this end, an in-depth search of peer-reviewed articles published until January 2025 was undertaken; no specific start date was used. Primary databases included PubMed/MEDLINE, Scopus, and Google Scholar. Search terms included combinations of “deep learning”, “artificial intelligence”, “intrahepatic cholangiocarcinoma”, “CNN”, and “computer-aided diagnosis”. We prioritized studies that specifically applied DL models to clinical data and radiologic or histopathologic imaging of iCCA; original research articles and high-quality reviews were included to support the interpretation. Reference lists of included papers were also screened for additional relevant articles.

Starting from a concise presentation of DL functional principles and evaluation metrics, published DL models applied to the diagnosis of iCCA using abdominal radiologic imaging and whole slide histopathology are presented. Recent approaches to predict the histopathologic features, survival, and recurrence are also highlighted. In addition, an analysis of the current limitations related to DL research as well as potential strategies to overcome these challenges are discussed.

**Table 1:** A summary of common metrics used for the evaluation of DL models’ performance. Abbreviations: TP: true positive; TN: true negative; FP: false positive; and FN: false negative.

Metric	Formula	Explanation	Limitations	References
Sensitivity	$\frac{TP}{TP+FN}$	Measures the proportion of TPs that are correctly identified by the model. Used in tasks where capturing all positive instances is essential, aiming to minimize FNs.	Recall is sensitive to dataset imbalance and may not be sufficient to assess the overall performance of a model.	[20,21]
Specificity	$\frac{TN}{TN+FP}$	Measures the proportion of TNs that are correctly identified by the model. Dual metric to sensitivity, being used in tasks where capturing all negative instances is essential, thus minimizing FPs.	Specificity can be less informative in highly imbalanced datasets. When the negative class is predominant, a biased model may overestimate specificity at the expense of low FN rates. Conversely, if the negative is underrepresented with few samples, specificity may fail to accurately reflect the model’s ability to identify TNs in real-world scenarios.	[20,21]
Accuracy	$\frac{TP+TN}{TP+TN+FP+FN}$	Calculates the proportion of correctly classified instances out of the total instances, offering an estimate of the model’s misclassification probability.	May be inadequate for imbalanced datasets and tasks where certain classes have greater significance. In the presence of class imbalance, a model biased toward the majority class can still achieve high accuracy. In cases where misclassifications have unequal consequences, accuracy treats all errors equally, failing to reflect the varying significance of different classes.	
Precision	$\frac{TP}{TP+FP}$	Quantifies the ratio of TP predictions to the total positive predictions made by the model. Used in tasks where minimizing FPs is a primary concern.	Sensitive to class imbalance, particularly when the positive class is significantly underrepresented. In such cases, precision is derived from a limited number of samples, making it unreliable. Conversely, when the positive class dominates, precision alone may not adequately reflect the FN rate, limiting its usefulness.	[21,22]
F1-score	$2\cdot \frac{Precision\cdot Recall}{Precision+Recall}=$ $\frac{TP}{TP+\frac{1}{2}\cdot (FP+FN)}$	Defined as the harmonic mean of precision and recall, offering a trade-off between the two. While precision focuses on minimizing FPs and recall on maximizing TPs, this balanced metric remains robust in imbalanced datasets and is particularly valuable in tasks where both FPs and FNs are important to consider.	Assigning equal weight to precision and recall in the F1-score may not be appropriate in imbalanced datasets, where minimizing the FPs or FNs may be more critical depending on the clinical context. In such cases, the F1-score may not adequately reflect the importance of these errors, as it treats both precision and recall equally without considering their relative significance in detecting the minority class.	[21,22]
Jaccard index	$\frac{TP}{TP+FP+FN}$	Seeks to capture all positive instances, while accounting for both FPs and FNs. Similar to F1-score, though it imposes a greater penalty on false predictions.	It assigns equal weight to FPs and FNs, which may not be appropriate for certain applications.	[21]
Area under the ROC curve (AUC)	There no general formula exists, as the shape of the AUC can differ across various applications. However, it can be calculated numerically given the TP and FP rate pairs.	The ROC curve is a graph of the TP Rate=g(FP Rate), computed across various discriminative thresholds. Since an optimal condition would involve a TP rate of 1 and an FP rate of 0, it can be concluded that a larger area under the ROC curve indicates a model design that is closer to optimal performance.	It may not be ideal for applications involving imbalanced datasets, where the minority class is of primary concern, as the ROC curve does not account for the different consequences of FPs and FNs. In such cases, sensitivity and specificity can have different levels of importance and the ROC curve may not fully reflect the impact of these errors, especially when the clinical decision relies on a precise trade-off between the two. Selecting appropriate discrimination thresholds for plotting the ROC curve can be challenging in imbalanced datasets, potentially masking the performance of the model on the minority class.	[21,22]

**Table 2:** Summary of studies testing DL models for iCCA diagnosis. Abbreviations: iCCA: intrahepatic cholangiocarcinoma; HCC: hepatocellular carcinoma; CNN: convolutional neural network; CT: computer tomography; MRI: magnetic resonance imaging; NR: not reported; STE: spatiotemporal excitation; DAM: difference attention module; AUC: area under the curve; STIC: Spatial Extractor-Temporal Encoder-Integration-Classifier; LilNet: liver lesion network; and H-LSTM: Hierarchical Long Short-Term Memory model.

Study Objective	Study Type	Type of Data	Imaging Modality	Model	Pretraining	Total Patients	Test Set Performance	Physician Comparison	Reference
ICCA and HCC classification	Single-center, retrospective	Images, tumor marker information	CT	Custom-made CNN	NR	617	Accuracy: 61.0% Sensitivity: 75.0% Specificity: 88.0%	Yes	[10]
ICCA-HCC classification	Single-center, retrospective	Images	CT	ResNet18 with STE module	ImageNet	398	Accuracy: 85.0% F1-score: 84.9% NPV: 88.2% AUC: 0.88	No	[23]
ICC-HCC classification	Two-center, retrospective	Images	CT	U-net with a DAM and a transformer network	NR	527	Accuracy: 81.6% Sensitivity: 73.4%, Specificity: 89.6% AUC: 0.86	No	[24]
Classification of HCC, ICCA, and CRLM	Multi-center, retrospective	Images	CT	InceptionV3	ImageNet	814	Accuracy: 96.2% Sensitivity: 93.7% Specificity: 98.5% PPV: 93.2% NPV: 88%	Yes	[11]
Automated diagnosis of focal liver tumors	Multi-center, retrospective	Images	CT	LilNet	ImageNet	4039	Accuracy: 88.7% AUC: 95.6% F1-score: 89.7% precision: 92.0% Sensitivity: 88.7%	Yes	[25]
ICCA and HCC classification	Multi-center, retrospective	Images	CT	H-LSTM	Yes	276	Accuracy: 91.0% Sensitivity: 91.0% Precision: 92.0% F1-score: 91.0% AUC: 93.0%	No	[26]
ICC-HCC, classification	Two-center, retrospective	Images and clinical data	CT	STIC	NR	723	Accuracy: 86.2% AUC: 89.0%	Yes	[27]
Reclassification of cHCC-CCA	Multi-center, retrospective	Images	WSI	ResNet50	TCGA	405	N/A	Yes	[28]
ICCA, HCC classification	Single-center, retrospective	Images	MRI	SFFNet	NR	112	Accuracy: 92.2% AUC: 96.8% Precision: 94.0% Sensitivity: 89.0% F1-score: 90.0%	No	[29]
Liver lesion classification	Single-center, retrospective	Images and clinical data	MRI	Inception-ResNetV2	ImageNet	1210	AUC: 89.7–98.7% Sensitivity: 53.3–100% Specificity: 91.6–99.5%	Yes	[30]
ICCA and HCC classification	Multi-center, retrospective	Images	MRI	Fusion VGG19 radiomics	ImageNet	381	AUC: 98.0% Accuracy: 87.5% F1-score: 88.0%	No	[31]
ICCA and HCC classification and ICCA grade prediction	Single-center, retrospective	Images	MRI	Fusion ResNet50 radiomics	ImageNet	162	AUC: 90.0% Sensitivity: 93.0% Precision: 96.0% F1-score: 91.0% Accuracy: 90.0%	No	[32]
Liver lesion classification	Single-center, retrospective	Images	MRI	Custom-made CNN	NR	296	Accuracy: 90.0% Sensitivity: 90.0% Specificity: 98.0%	Yes	[33]
ICCA, HCC, and cHCC-CCA classification	Single-center, retrospective	Images	US	ResNet18	NR	465	AUC: 92.0% Sensitivity: 84.6% Specificity: 92.7% Accuracy: 86.0% PPV: 85.5% NPV: 93.0% F1-score: 85.0%	No	[34]
Liver lesion classification	Multi-center, prospective	Images, histopathological biomarkers, and clinical data	US	Long Short-Term Memory, multilayer perceptron	NR	3342	Accuracy: 86% Specificity: 97% Sensitivity: 85% Precision: 81% NPV: 97% F1-score: 83%	Yes	[12]
ICCA, HCC, and cHCC-CCA classification	Single-center, retrospective	Images	WSI	ResNet18	ImageNet	161	Diagnostic agreement for HCC: 96.0%, ICCA: 87.0%	No	[35]

**Table 3:** Summary of studies testing DL models for histopathological feature prediction. Abbreviations: iCCA: intrahepatic cholangiocarcinoma; CNN: convolutional neural network; NR: not reported; MVI: microvascular invasion; OCT: optical coherence tomography; and AUC: area under the curve.

Study Objective	Study Type	Type of Data	Imaging Modality	Model	Pretraining	Total Patients	Test Set Performance	Physician Comparison	Reference
Preoperative prediction of MVI	Multicenter, retrospective	Images	MRI	MFCNN	NR	519	AUC: 88.0% Accuracy: 86.8% Sensitivity: 85.7% Specificity: 87.0%	No	[15]
Prediction of pathological differentiation	Single-center, prospective	Images	CT	ResNet50, SeNet50, DenseNet50	NR	408	AUC: 64.0–65.0% Accuracy: 68.0–68.6%	No	[16]
Ex vivo differentiation of iCCA and liver parenchyma	Single-center, prospective	Images	OCT	Xception	NR	11	F1-score: 94.0% Sensitivity: 94.0% Specificity: 93.0%	No	[18]

**Table 4:** Summary of studies testing DL models for survival prediction. Abbreviations: CT: computer tomography; NR: not reported; AUC: area under the curve; OS: overall survival; PFS: progression-free survival; ML: machine learning; DL: deep learning.

Study Objective	Study Type	Type of Data	Imaging Modality	Model	Pretraining	Total Patients	Test Set Performance	Physician Comparison	Reference
Survival prediction	Single-center retrospective	Images, clinical data, genomic profiling	WSI	ResNet	NR	83	Concordance index: 0.80 (OS), 0.72 (PFS)	No	[36]
Early postoperative recurrence	Multicenter retrospective	Images	CT	ResNet50	No	41	AUC: 99.4% Sensitivity: 97.8% Specificity: 94.0% PPV: 96.7% NPV: 96.1%	No	[14]
Preoperative identification of high-risk patients for futile surgery	Multicenter retrospective	Demographic data, clinical data, imaging data	NR	Ensemble ML-DL model	NR	827	AUC: 78.0% Sensitivity: 64.6% Specificity: 80.0% PPV: 73.1% NPV: 72.7%	No	[13]

## 2. DL for the Diagnosis of iCCA

iCCA and HCC constitute the two most common primary liver malignancies. Differentiating iCCA from HCC can be a diagnostic challenge due to overlapping imaging characteristics [37]. Accurate diagnosis is essential as the treatment strategy and prognosis differ substantially [38]. Traditional imaging modalities provide radiologic features that may not always provide a definitive diagnosis. In recent years, artificial intelligence using DL methods for image analysis has emerged as a powerful adjunct to enhance the diagnostic accuracy in liver tumor imaging [39].

### 2.1. Computer Tomography (CT)

Nakai et al. reported two DL models using CNNs that outperformed radiologists to distinguish between moderately differentiated HCC (mHCC), poorly differentiated HCC (pHCC), and iCCA based on triple-phase CT images [10]. In this study, two distinct models were developed: a one-input model using only images as input and a two-input model using images and tumor marker information. A total of 617 patients were enrolled and allocated into training, validation, and test sets with an 80:10:10 split. A histopathological diagnosis was used as the gold standard diagnosis. The two-input model achieved an overall classification accuracy of 61% versus 60% for the one-input model versus 55% and 53% for each of the two radiologists, respectively. Interestingly, the two-input model was inferior to radiologist 1 in terms of sensitivity (75% vs. 92%) and specificity (88% vs. 90%) in differentiating between iCCA versus HCC, while this model was superior in differentiating between pHCC versus mHCC (specificity: 68% vs. 45%, respectively). While the addition of tumor marker information resulted in a slightly enhanced accuracy, the performance of the two-input model was no better in classifying iCCA and HCC. In fact, the model that incorporated tumor marker data was even slightly worse in differentiating between pHCC and mHCC (sensitivity: 62% vs. 64%; specificity of 68% vs. 70%, respectively), highlighting the more important role of imaging features to make a diagnosis.

Xue et al. proposed a CNN based on a spatiotemporal excitation (STE) module for iCCA and HCC recognition using CT images [23]. The authors employed ResNet18 [40] as the basic network and inserted an STE module in each residual block. ResNet18 is a specific configuration within the Residual Network (ResNet) family, which was designed to address the challenges of training very deep networks, particularly vanishing gradients [40]. ResNet introduced a residual learning framework, which involved residual connections that bypass one or more layers and feed the output of one layer as the input to the next layers. This innovation allows gradients to propagate more effectively through the network by enabling the model to learn residual functions, instead of direct mappings, and facilitates the training of deeper architectures without performance degradation. ResNet is available in several configurations with differing depths, each providing a trade-off between model complexity, training efficiency, and task performance. ResNet18 is a relatively shallow network, with fewer layers and parameters compared with deeper versions, which have lower computational demands and faster training at the expense of lower accuracy. The STE module consisted of two components: a spatial excitation (SE) module that calculates differences between consecutive CT slices to emphasize shape-sensitive features, while the temporal interaction (TI) module shifts channels in the temporal dimension to enable information exchange between adjacent CT slices. The weights of ResNet18 pretrained on ImageNet were utilized [41]. The authors performed channel expansion by adjusting the window width and window level to generate two additional images, which were combined with the original image to form a three-channel image. This approach improves the richness of the feature representation, making lesions more pronounced and improving the model’s ability to capture and learn more discriminative imaging features. CT images from 398 patients were collected and assigned into a training and test set with a 4:1 ratio. Manual annotation was performed by a single experienced radiologist. ResNet18 with the STE module demonstrated a superior classification performance for classifying HCC versus iCCA compared with a 3D CNN module or ResNet plus either the SE or TI modules alone, achieving an average accuracy with an area under the ROC curve (AUC), F1-score, and negative predictive value (NPV) of 0.85, 88.9%, 84.9%, and 88.2%, respectively. The classification performance of the proposed DL model was consistently better, based on all evaluation metrics, on multichannel images compared with single-channel images, confirming the effectiveness of channel expansion to improve lesion detection.

Wu et al. [24] proposed a DL-based phase difference network for the differentiation of iCCA and HCC based on a four-phase CT, which outperformed several other published DL models [27,42,43,44]. The proposed model was built using a U-Net enhance encoder for contrast adjustment, a difference attention module (DAM), and a transformer network as the classification module. The DAM was used to examine the interphase temporal information and enhance the feature representation; the classification module was used to assess the overall context and detect interphase temporal relationships. A total of 398 cases were used for model training after data augmentation. The proposed model demonstrated a superior classification performance across all selected evaluation metrics (accuracy 81.66%, sensitivity 73.4%, specificity 89.6%, and AUC 86%) versus MSCS-DeepLN [43], STIC [27], modified SeNet [44], as well as the CNN architecture proposed by Oestmann et al. [42] Subsequently, the model demonstrated an excellent generalization capacity upon external validation, while still maintaining its performance superiority over comparator models. In addition, based on ablation experiments, the complete network consistently outperformed variants without the enhance encoder or the DAM.

Midya et al. presented a DL model based on the InceptionV3 architecture [45] for the automated classification of HCC, iCCA, and CRLM based on CT images [11]. The established model demonstrated a greater classification accuracy compared with two experienced radiologists (96.27% vs. 92.55% and 84.47%, respectively), while also being consistently superior in terms of sensitivity, specificity, and the positive and negative predictive value. Importantly all cases misclassified by both radiologists were accurately classified by the DL model. Furthermore, the proposed model markedly outperformed other DL models including VGG [46], ResNet [40], and DenseNet [47].

Wei et al. designed a DL model based on the YOLOV8 architecture [48], termed the Liver Lesion Network (LiLNet), for the automated diagnosis of focal liver lesions based on multiphase CT images [25]. Three variations of the LiLNet model were trained to distinguish between benign and malignant disease, different types of malignant tumors, and different types of benign lesions. Within the test set, LiLNet achieved an AUC, accuracy, F1-score, recall, and precision ranging from 0.95 to 0.97, 88.6% to 94.7%, 89% to 94.9%, 88.4% to 94.7%, and 89.9% to 95.2%, respectively. During the external validation using image data from 1151 patients from four different centers, LiLNet demonstrated an excellent generalizability with an AUC, accuracy, F1-score, recall, and precision ranging from 0.87 to 0.94, 74.4% to 90%, 81.6% to 90%, 80.8% to 89.9%, and 83.6% to 90.1%, respectively. The background liver condition and tumor size had a minimal impact on the model’s performance. Within the benchmarking image test set (n = 6743), the DL model had a greater diagnostic accuracy compared with junior-, middle-, and senior-level radiologists in differentiating benign from malignant tumors (91.0% vs. 76.4%, 76.9%, and 78.7%, respectively), as well as in the diagnosis of benign lesions (92.3% vs. 72.3%, 82.3%, and 85.4%, respectively). The classification performance of the model was comparable to that of radiologists for malignant tumors.

Huang et al. designed a Hierarchical Long Short-Term Memory model using ResNet18 [40] to automate the distinction of HCC, iCCA, and normal parenchyma based on four-phase CT images [26]. CT data from 276 individuals were collected and allocated into training, validation, and testing sets at a 7:1:2 ratio. ResNet18, pretrained using ImageNet, demonstrated a superior performance compared with the DenseNet [47], InceptionNet [45], and EfficientNet [49] models and a comparable performance to various types of ResNet [40] and VGGNet [46] architectures. The proposed model achieved a three-class classification accuracy of 91.0%. Subsequently, the model was tested using varying combinations of two, three, or four CT phases. The combination of plain, arterial, and delayed phases achieved the best results for iCCA identification (AUC of 0.90).

Gao et al. developed a DL model with a modular design of the Spatial Extractor-Temporal Encoder-Integration-Classifier (STIC) for the classification of malignant liver lesions based on multi-phase CT images and clinical data [27]. The STIC model achieved an AUC of 89% and an accuracy of 86.2% for the classification of HCC versus iCCA within the test set, outperforming two benchmark models using the channel assignment strategy. Subsequently, the model was evaluated for the classification of primary and secondary malignant hepatic tumors; the model achieved an AUC of 0.86 and an accuracy of 72.6% compared with an overall accuracy of 70.8% based on a radiologist consensus. Importantly, the radiologist diagnostic performance was significantly enhanced with the assistance of the STIC model, reaching 79.1%. Within the external test set, the STIC model demonstrated a good generalizability, achieving an accuracy of 82.9% and an AUC of 0.94.

Calderaro et al. developed a DL model using ResNet50 [40] that effectively reclassified cHCC-CCA as pure HCC or iCCA based on whole slide images (WSIs) [28]. The model underwent self-supervised pretraining using image data from The Cancer Genome Atlas (TCGA). The model was tested on two patient cohorts with histopathologically proven HCC and iCCA from different centers. The model demonstrated an excellent classification performance, achieving an AUC of 0.99 and 0.94. Subsequently, the model was evaluated using a multicentric cohort comprising 405 cases. The model correctly assigned regions with an HCC- or iCCA-like morphology based on high “HCCness” and “iCCAness”, respectively. Only a slight concordance was observed between the pathological reclassifications made by an expert liver pathologist and the model prediction (Cohen’s Kappa: 0.19). Importantly, cases reclassified by the model as iCCA had a shorter median overall survival after either resection or liver transplantation compared with patients reclassified as HCC. In contrast, the conventional reclassification was not associated with the prognosis. Furthermore, the model’s prediction matched the genomic alterations of cHCC-iCCA, as all genomic alterations in HCC-specific genes and 11 out of 16 alterations typically noted in iCCA were in the subset of patients reclassified as HCC and iCCA, respectively.

### 2.2. Magnetic Resonance Imaging (MRI)

Liu et al. developed a novel residual DL model to differentiate mass-forming iCCA versus HCC based on MRI [29]. This model, termed the strided feature fusion model (SFFNet), comprises a multilayer fusion module, a stationary residual block (SRB), and an convolutional block attention module (CBAM). The authors employed ResNet101 [40] as the base network model and the ImageNet database [41] for model pretraining. They introduced an SRB aimed to optimize SFFNet’s performance, making it more effective and generalizable compared with traditional ResNet101. While reducing redundant normalization can improve computational efficiency, batch normalization also helps to control the feature scale and stabilizes training, especially in deeper networks like ResNet101. The addition of the CBAM further was aimed at improving the model’s performance via the incorporation of spatial and channel-wise attention mechanisms. The CBAM refines feature maps by sequentially applying channel attention followed by spatial attention. The channel attention module prioritizes important feature channels by adaptively recalibrating their weights, helping the network focus on the most meaningful information in the image. The spatial attention module enhances the network’s ability to identify key regions by refining the spatial distribution of features. This dual refinement improves the model’s capability to capture fine-grained details, making it particularly beneficial for complex imaging tasks. A total of 2207 images were used in the training and validation sets after data augmentation. SFFNet versus ResNet101 and ResNet101 plus either CBAM, SRB, or MFF alone were compared. SFFNet was consistently superior relative to all evaluation metrics, achieving an accuracy of 92.2%, an AUC of 0.96, as well as a precision, recall, and F1-score for MF-iCCA of 94.0%, 86.0%, and 90%, respectively. Additionally, this model outperformed other widely used classification models including AlexNet [50] (accuracy: 79%, AUC: 0.86), VGG19 [46] (accuracy: 79%, AUC: 0.86), DenseNet169 [47] (accuracy: 76%, AUC: 0.83), EfficientNet [49] (accuracy: 73%, AUC: 0.80), and Inception V3 [45] (accuracy: 78%, AUC: 0.88).

Zhen et al. reported a DL model based on Inception-ResNetV2 [51], pretrained on the ImageNet database, that demonstrated a superior performance to three experienced radiologists in the classification of liver cancers based on MRI images [30]. Inception-ResNetV2 is a hybrid DCNN architecture that integrates residual connections into the Inception architecture [51]. Given that Inception networks tend to be very deep, this hybrid variant significantly improves the recognition performance as it combines the feature extraction capabilities of Inception modules with the training benefits of residual connections. A training set was used, which comprised 31,608 MRI images from 1210 patients including images from six different scan sequences, to achieve a seven-way classification diagnosis (cyst, hemangioma, focal liver lesion, other benign nodules, HCC, metastases, and non-HCC primary liver cancer), binary classification (benign lesion and malignancy), and three-way malignancy classification (HCC, metastases, and non-HCC primary). A diagnosis based on histopathology was used as the ground truth. The validation cohort comprised 6,816 images from 201 individuals. Two distinct models trained with only unenhanced sequences or enhanced plus unenhanced demonstrated a comparable performance to diagnose malignant conditions (AUC: 0.946 and 0.951 and sensitivity: 90.9% and 91.9%, respectively). Three different models that utilized clinical data plus six or three imaging sequences or images alone, respectively, were tested relative to the task of a three-way malignancy classification. Classifiers that combined clinical data and images demonstrated an excellent performance, which outperformed all other models and expert abdominal radiologists (accuracy: 93.9% for the six-sequence model, 91.9% for the three-sequence model versus 88.1%, 77.1%, and 84.6% for the radiologists, respectively).

Wu et al. developed radiomics, DL models, and fusion DL-based radiomics for the preoperative discrimination of dual-phenotype HCC, HCC, and iCCA using MRI images [31]. The researchers pretrained the ViT [52], VGG19 [46], Googlenet, and InceptionV3 [45] models on ImageNet and subsequently fused radiomics and DL model features to develop 12 fusion models. VGG19 is a 19-layer-deep CNN with a deep hierarchical structure that was a top-performing model in the ImageNet Challenge. VGG19 has demonstrated a good generalizability to other datasets and is widely used in computer vision tasks. ViT is a DL model that employs a transformer architecture by dividing images into fixed-size patches and processing these images as sequences. ViT learns spatial relationships through self-attention, enabling it to capture long-range dependencies. Data inputs for the DL and fusion models were arterial phase images, portal phase images, or combined images. The study recruited 381 patients from four different centers. A total of 3,810 images were split into a training set (n = 2440), internal testing set (620), and external testing set (n = 750). Among the DL models, VGG19-combined demonstrated the highest classification accuracy (AUC: 0.94, accuracy: 79.7% and F1-score: 79%). The Fusion-VGG19-combined model emerged as the best-performing classifier, demonstrating an excellent diagnostic performance in the external testing set (AUC of 0.98, accuracy of 87.5%, and F1-score of 88%).

In a similar study, Wang et al. constructed a DL-radiomics model to classify HCC and iCCA, as well as predict the iCCA grade based on MRI images [32]. A total of 162 patients were enrolled and were randomized into a training set and a validation set at a 7:3 ratio. Five CNNs (ResNet50, VGG16, EfficientNet, InceptionV3, and ConvNet [53]) pretrained using the ImageNet dataset were trained and their classification performance was compared. ResNet50 attained the highest accuracy (79%) and was selected as the base model for the convolution feature extraction. Subsequently, radiomics and convolution features were fused and the SVM classifier was employed to develop the model. The fusion model demonstrated a superior performance compared with traditional radiomics, achieving an AUC, sensitivity, precision, F1-score, and accuracy of up to 0.90, 93%, 96%, 91%, and 90%, respectively. Compared with the standard region of interest, the 6 mm extended region resulted in the best prediction performance. The DL model using the 6 mm extended region of interest demonstrated an excellent predictive ability, achieving an AUC of 0.89, an F1-score of 93%, a sensitivity of 93%, and a precision of 93%.

Hamm et al. developed a custom CNN-based DL system for the automatic classification of liver tumors, including HCC, iCCA, cysts, hemangiomas, focal nodular hyperplasia, or CRLM, based on multi-phasic MRI images [33]. The study population comprised 296 patients with 494 lesions, which were allocated to training (n = 434) and test (n = 60) sets. The final training set comprised 43,400 images after the data augmentation through image processing techniques. In the test set, the established model achieved an accuracy of 90% for lesion classification, outperforming two experienced radiologists (accuracy: 80% and 85%, respectively). In a subsequent publication from the same research group, the model was fairly consistent to determine the presence and location of radiological features related to hepatic lesions, as well as their contribution to the lesion classification [54]. This proof-of-concept study related to an interpretable DL system was of particular importance as it provided insights into the decision-making process behind automated classification, partly resolving the “black-box” nature of DL models. Furthermore, this approach allows for precise fine-tuning through extended training based on features that the model fails to detect, as well as for the critical interpretation of the model’s output

### 2.3. Ultrasound (US)

Chen et al. constructed four DL models for the preoperative differentiation of HCC, iCCA, and combined hepatocellular cholangiocarcinoma (cHCC-CCA) based on B-mode ultrasound (US) images [34]. The proposed models were built using four different architectures: ResNet18 [40], MobileNet [55], DenseNet121 [47], and Inception V3 [45]. MobileNet is optimized for mobile and edge devices using depthwise separabale convolutions to reduce the computational cost while maintaining accuracy. DenseNet121 enhances feature reuse through dense connections in which each layer receives input from all previous layers, improving efficiency and reducing the number of parameters. Inception V3 employs a multi-scale feature extraction using Inception modules, which is effective for complex image recognition, yet computationally heavier than other approaches. A total of 465 patients were enrolled and allocated into a training (n = 330), validation (n = 85), and test set (n = 50). Before training, the data augmentation was applied to expand the number of images due to the small-scale dataset. ResNet18 achieved a greater overall validation accuracy (99.35% versus 75.03% for MobileNet, 53.35% for DenseNet121, and 40.77% for Inception V3). ResNet18 demonstrated similar diagnostic power in the independent test cohort, yielding an overall AUC of 0.92 and a sensitivity, specificity, accuracy, PPV, NPV, and F1-score of 84.6%, 92.7%, 86%, 85.9%, 93%, and 85%, respectively.

Ding et al. developed several models based on Long Short-Term Memory neural networks and a multilayer perceptron for the classification of focal liver lesions using US images, histopathological biomarkers, and clinical data; this model performed significantly better than junior radiologists among the 3342 enrolled patients enrolled [12]. The model was based on biomarker and US data and outperformed models using clinician-reviewed imaging plus clinical or biomarker data, achieving an accuracy of 90%, an AUC of 0.89, a specificity of 98%, a recall of 82%, an NPV of 98%, and an F1-score of 84%. Subsequently the model was tested prospectively and achieved an accuracy, specificity, and sensitivity of 86%, 97%, and 85%, respectively, which was better than junior radiologists and the same as senior US or senior MRI radiologists. Interestingly, the use of this model as an adjunct improved the performance of junior radiologists (accuracy increases of 24%, 15%, and 21%). Notably, senior radiologists plus AI did not lead to an improved performance (accuracy increases of 3%, 0%, and 3%, respectively).

### 2.4. Histopathology

Beaufrere et al. proposed a weakly supervised DL model using ResNet18 for the automated classification of primary liver malignancies, such as iCCA, HCC, or cHCC-CCA, based on WSIs [35]. The model was pretrained using ImageNet [41]. A total of 166 WSIs were reviewed by two liver pathologists who annotated tumor and non-tumor areas without a detailed tumor-type annotation. These images were subsequently allocated into a training set (n = 90), as well as internal (n = 29) and external (n = 47) validation sets. Through this weakly supervised learning method, the model successfully retrieved tumor-specific morphological features and demonstrated promising results in the unsupervised clustering and classification of liver tumors. In the internal validation set, the agreement between the pathological diagnosis and the model prediction was 78% for iCCA and 100% for HCC. Furthermore, the agreement between the immunohistochemical diagnosis and model predictions was 100% for HCC, 86% for iCCA, and 75% for cHCC-CCA. These outcomes were reproduced in an external validation set as the diagnostic agreement was 96% for HCC, 87%, for iCCA, and 83% for cHCC-CCA.

## 3. DL for Histopathological Feature Prediction

Gao et al. reported a multiparametric fusion (MFCNN) and a late fusion model for the preoperative prediction of microvascular invasion based on MRI images [15]. The MFCNN allows for end-to-end learning by processing and fusing features at an intermediate stage before the final decision layer. This approach enables the model to jointly optimize its parameters during training, learning the best way to integrate the different features for an improved performance. In contrast, in late fusion models, each modality is processed in an independent CNN branch, and the fusion happens at the decision level after processing is complete. Seven sequences of MRI were used as inputs and were passed into a five-layer CNN. The study enrolled 519 patients who were allocated into a training (n = 361), validation (n = 90), and external testing (n = 68) cohort. The MFCNN model was slightly superior to the late fusion model in the testing set, achieving an AUC, accuracy, sensitivity, and specificity of 0.88, 86.8%, 85.7%, and 87% versus 0.86, 83.8%, 78.6%, and 85.2%, respectively. Statistical significance was not reached for any of the evaluation metrics. Furthermore, the MFCNN outperformed all seven monoparametric CNN models using a single MRI sequence as the input. The CNN model using arterial phase images performed the best among the seven models, with an AUC of 0.86 in the testing set.

Wolff et al. employed the Xception CNN architecture for the differentiation of ICCA and liver parenchyma ex vivo using optical coherence tomography (OCT) as a means of a noninvasive assessment of resection margins [18]. OCT relies on low-coherence interferometry to capture real-time, high-resolution cross-sectional images of biological tissues [56]. Xception (Extreme Inception) is a DCNN in which Inception modules have been replaced by depthwise separable convolutions [57]. Xception builds on the idea that spatial and channel-wise feature extraction can be fully decoupled leading to performance gains via the more efficient use of model parameters. Xception outperformed Inception V3 on large datasets [57]. A total of 24 OCT scans from 12 tumor areas and 12 areas of healthy parenchyma, comprising 85,603 images, were used. Data were split into a training, validation, and test set at a 70:15:15 ratio. The proposed model achieved a mean F1-score, sensitivity, and specificity of 94%, 94%, and 93%, respectively.

Xia et al. developed three DL models using a re-weight method (SoftMax Equalization Loss, SEQL) for pathological differentiation predictions using CT images [16]. The study population comprised 408 patients who were randomized into a training set (n = 328) and a test set (n = 80). Three different types of architectures were used for the model development, including ResNet50, SeNet50, and DenseNet50; re-weighting was used to mitigate the impact of class imbalance. All three models had a comparable performance for predicting the degree of differentiation (low or medium-high), achieving an AUC and an accuracy ranging from 0.64 to 0.65 and from 68% to 68.6%, respectively. Importantly, in ablation experiments, models developed using the SEQL method consistently outperformed baseline models. Furthermore, an ensemble model, using multi-model ResNet50, SeNet50, and DenseNet121, exhibited a superior accuracy compared with individual models (accuracy of 72.5%), indicating the performance-enhancing effect of model integration.

## 4. DL for Prediction of Recurrence and Survival

Xie et al. developed a DL-based approach to predict survival based on WSIs of iCCA patients [58]. A U-net model was used for multi-tissue segmentation, and an AlexNet-based DCNN model was used for lymphocyte detection. U-net is a CNN model developed for segmentation tasks, utilizing an encoder–decoder architecture with skip connections to achieve precise localization [59]. The encoder extracts hierarchical features, progressively reducing the spatial resolution while capturing essential patterns. The decoder then upscales these features restoring the spatial resolution and assigning a label to each pixel. The study enrolled 127 patients with histopathologically confirmed iCCA; 78 patients were allocated into the training set, and 49 cases comprised the testing set. The model exhibited an excellent performance in the delineation of liver parenchyma, tumor lesions, necrosis, or other tissues with an overall DICE coefficient of 0.86. For lymphocyte detection, the model achieved an F1-score of 82%, a precision of 88%, and a recall of 77%. Subsequently, three ML techniques, including Linear Discriminant Analysis, Quadratic Discriminant Analysis, and a Decision Tree classifier, were used to construct the binary survival estimate model. This image-based model emerged as an independent predictor of patient survival (HR: 2.90, 95%CI: 1.35–6.89); however, the image-based model was not an independent predictor of recurrence (HR: 1.89, 95%CI: 0.78–4.59).

Schmauch et al. designed a model using ML classification techniques and a ResNet feature extractor to predict patient outcomes after surgery for iCCA using clinical data, WSIs, and genomic profiling [36]. A total of 83 patients were enrolled and one to three WSIs from each patient were used. A model based on clinical and WSI data achieved a concordance index for OS and PFS of 0.74 and 0.73, respectively. Genetic profiling information further improved the performance for OS (0.80) but not PFS (0.72). Both models demonstrated superior performances compared with the patient stratification based on TNM staging alone (0.66 and 0.59, respectively).

Wakiya et al. constructed a robust DL model using ResNet50 to predict the early postoperative recurrence of iCCA based on CT images [14]. The model did not undergo pretraining. A total of 71,081 patches from 41 patients treated in three centers were used and allocated into training and validation datasets at a 5:1 ratio. The model demonstrated a very good prediction performance in the validation dataset, achieving an AUC of 0.994, a sensitivity of 97.8%, a specificity of 94.0%, as well as positive and negative predictive values of 96.7% and 96.1%, respectively. A comparison of the patient and tumor characteristics between cases with prediction accuracies below and above 96% identified a smaller tumor size as a significant factor contributing to misprediction.

Altaf et al. developed several AI models using ML and/or DL algorithms for the preoperative identification of patients at high risk of undergoing futile surgical resection [13]. A total of 827 patients were retrospectively enrolled and randomized into a training and test cohort using an 8:2 split ratio. Ten preoperative factors that were associated with a futile resection on univariable analysis were selected for model training. Among the developed models, an ensemble model combining a gradient boosting machine classifier and a multilayer perceptron emerged as the best performing, achieving an AUC of 0.78, a sensitivity of 64.6%, a specificity of 80%, and a positive and negative predictive value of 73.1% and 72.7%, respectively.

## 5. Limitations and Future Perspectives

The development and integration of DCCNs into standard medical practice is a topic of debate given the technical and interpretability issues involved in model training and employment. Due to their inherent complexity, DL models require prolonged training cycles and access to extensive, high-quality datasets to develop an accurate pattern recognition and robust in-sample and out-of-sample performance [60]. High-quality datasets must meet certain key criteria, including high-resolution images, sufficient diversity, balanced data distribution, a large sample size, a lack of bias, and precise image annotation [8].

Annotating specialized medical images poses several challenges that impact the accuracy and efficiency of the model development. Manual annotation with ground truth is a labor-intensive process that demands specialized knowledge and cross-validation among experts to compensate for interobserver variability and reduce labeling errors (labeling bias). Acquiring large datasets of annotated medical images is particularly challenging in specialized applications in which the scarcity of data constitutes a significant obstacle to model development and generalizability. To mitigate these challenges several strategies have been proposed. Transfer learning is a widely adopted approach that allows models trained on one task to be repurposed and perform on a new, related task. By leveraging pre-existing knowledge, transfer learning reduces the need for extensive new training and the corresponding computational resources [61]. In another paradigm, self-supervised DL models are trained with the non-annotated part of the input data for which auto-generated labels are created. By learning representations from unlabeled data, DL models are able to learn the rest of the data, effectively transforming unsupervised learning into supervised learning [62]. Notably, transfer learning is a key enabler of foundation models (FMs). FMs are large-scale models pretrained on massive, diverse datasets, generally employing self-supervised learning, designed to be versatile and adaptable across a wide range of downstream tasks with minimal task-specific tuning [63]. FMs have produced promising results in medical tasks; however, further research is needed to address the issues of data privacy, interpretability, validation, and verification before their integration into clinical workflows [63]. Interestingly, federated learning offers a promising approach to training DL models while preserving patient data privacy. Federated learning enables multiple healthcare providers to train a model collaboratively without the need to share actual patient data [64]. Model updates are shared allowing for the accumulation of data from different clinical settings. By enabling the use of decentralized data, this approach could be particularly valuable in iCCA, where data scarcity makes model design and generalization difficult.

Data augmentation techniques, such as image rotation, flipping, scaling, and noise addition, artificially expand training datasets with variations in original images, which—while sharing similar features—are perceived as alien data by the DL model, supplementing the effective training set volume and improving the model robustness. Models’ exposure to modified images, which can be more representative of real-world image quality, increases the generalizability of the model’s performance in real-world scenarios [65].

In addition, publicly available, large-scale annotated datasets are increasing in number and improving in quality and can be leveraged to augment pretraining or training data effectively beyond the image acquisition capacity of individual research groups. However, there exists a need for datasets of specialized images relevant to the task at hand that can be used for domain-specific pretraining. Domain-specific pretraining involves initializing models using input from the target domain, resulting in representations that capture the domain’s unique statistical properties and semantic structures. In the discussed studies, the model pretraining relied on publicly available datasets, such as ImageNet, which may not optimally represent the complexities of liver tumor imaging. To this end, Struyvenberg et al. demonstrated that a model pretrained using domain-specific images performed better versus pretraining with ImageNet or a lack of pretraining (accuracy of 83% versus 82% and 75%, respectively) [66]. Further research is warranted to explore the impact of domain-specific pretraining and to construct such datasets. Given the advent of minimally invasive surgery for iCCA [67], this will be of particular importance for developing highly effective DL models for intraoperative surgical guidance.

Furthermore, it should be emphasized that the stand-alone performance of DL models does not equal clinical utility, and careful attention is required to interpret outcomes and extrapolate DL models in specific clinical scenarios.

The currently published studies had certain inherent limitations. The majority of studies were retrospective and most of them were conducted in tertiary centers. These controlled conditions often failed to reflect the variability and complexity of real-world clinical practice, in which differences in operator expertise, imaging protocols, and patient populations introduced challenges to the external validity. DL models are highly task-specific, and their performance is tightly constrained by the nature of the training dataset as the models cannot extrapolate beyond the patterns encountered during training. Accordingly, it is essential that the clinician interpreting and applying DL systems is aware of the biases that may hinder the generalizability of the model’s performance to their patient population.

To ensure clinical applicability, DL models designed for the primary detection of iCCA should be evaluated on data that are representative of the average-risk population. Otherwise, selection bias can arise, leading to a suboptimal performance when applied in non-specialized centers or lower-prevalence populations. Selection bias occurs when datasets are not representative of the general population, which is often due to non-randomly distributed data sourcing from specialized centers. Many DL studies in medical image analysis rely on datasets collected from specialized, high-volume centers with state-of-the-art imaging equipment and may not capture the variability in the image quality encountered in everyday clinical practice. Additionally, these datasets may contain a disproportionately high number of diseased cases, leading to predictive values that do not translate effectively to settings with a lower disease prevalence. Spectrum bias may also arise in cases that use only images that are clearly representative of iCCA, leading to a suboptimal performance on atypical presentations. This can lead to models that perform well in controlled research settings but fail to be generalizable across diverse patient populations, non-specialist settings, and varying equipment qualities. Strategies to mitigate selection bias and generate more diverse patient cohorts include Generative Adversarial Networks (GANs), which can generate synthetic examples of underrepresented cases [68]. Furthermore, in the training set, cases can be oversampled or underweighted relative to how frequent the cases occur in real-world settings to reflect the disease prevalence more accurately.

Despite these technical limitations, perhaps the most pressing challenge restricting the widespread clinical adoption of DL models is the lack of interpretability. The decision-making mechanisms employed by such deep networks, including both the feature extraction and discriminative stage, cannot be decoded by humans. This fact proves to be problematic in two senses. First, there are no robust mathematical guidelines to confidently recognize what elements of the DL architecture lead to a particular output behavior. As a result, the tuning of the model is based almost exclusively on heuristics, rather than theoretically supported arguments. Secondly, from an end-user’s perspective, the inference process performed by a model to produce an output prediction cannot be broken down into verbally expressed reasoning, as would be the case with a human expert performing the same task, and is therefore impossible to follow. This “black-box” nature raises concerns when DL-driven diagnoses or predictions contradict expert opinions, especially in high-stakes medical decisions—creating barriers for DL integration into routine clinical practice. The demand for more transparent and interpretable models has fueled interest in explainable AI, which seeks to develop methods providing insights into how models reach their conclusions. Techniques such as saliency maps, class activation mapping, and attention mechanisms help visualize the decision-making process. Despite advancements, fully interpretable DL models remains a distant possibility [69]. Another fundamental, yet often overlooked, challenge is the need for technical literacy. Clinicians should commit to developing a foundational understanding of the functional principles and key concepts of DL and attain an adequate familiarity with the technical terminology. An effective DL integration into medical workflows goes beyond passive adoption and demands active engagement from physicians who can critically assess reliability and contextualize and refine AI-generated insights. Bridging the knowledge and communication gap between physicians and AI professionals, as well as enabling interprofessional and doctor–machine communications [70], is vital for its optimal use by the clinicians and for the model performance optimization via bias minimization and an alignment with medical standards.

## 6. Conclusions

The demonstrated success and approval of computer-aided diagnosis systems for the real-time interpretation of liver lesions highlight the potential of DL models across various medical imaging applications. For iCCA, several studies have reported highly accurate DL models for diagnosis based on abdominal radiologic exams, which demonstrate comparable or superior performances compared with radiologists. These approaches hold potential as adjuncts for the abdominal radiologist, leading to a more accurate diagnosis, an optimal therapeutic strategy, and improved outcomes. Despite the influx of high-quality studies, no DL models have received regulatory approval from the FDA for routine clinical use in iCCA. Although the available evidence is promising, the transition of DL models from research settings to routine clinical practice requires overcoming several technical and practical barriers. Ultimately, the future of DL in medicine depends not only on technological advancements but also on the ability of clinicians to harness these tools effectively. In the future, DL will serve as an enhancement to domain-specific medical decisions, streamlining the diagnostic process and optimizing patient outcomes.

## Data Availability

Data supporting the recommendations of this article are included within the reference list.
